# Gammaherpesvirus infection and malignant disease in rhesus macaques experimentally infected with SIV or SHIV

**DOI:** 10.1371/journal.ppat.1007130

**Published:** 2018-07-12

**Authors:** Vickie A. Marshall, Nazzarena Labo, Xing-Pei Hao, Benjamin Holdridge, Marshall Thompson, Wendell Miley, Catherine Brands, Vicky Coalter, Rebecca Kiser, Miriam Anver, Yelena Golubeva, Andrew Warner, Elaine S. Jaffe, Michael Piatak, Scott W. Wong, Claes Ohlen, Rhonda MacAllister, Jeremy Smedley, Claire Deleage, Gregory Q. Del Prete, Jeffrey D. Lifson, Jacob D. Estes, Denise Whitby

**Affiliations:** 1 AIDS and Cancer Virus Program, Leidos Biomedical Research, Frederick National Laboratory for Cancer Research sponsored by the National Cancer Institute, Frederick, Maryland, United States of America; 2 Pathology/Histotechnology Laboratory, Leidos Biomedical Research, Frederick National Laboratory for Cancer Research sponsored by the National Cancer Institute, Frederick, Maryland, United States of America; 3 Laboratory of Pathology, Center for Cancer Research, NCI, Bethesda, Maryland, United States of America; 4 Vaccine and Gene Therapy Institute, Oregon Health & Sciences University, Beaverton, Oregon, United States of America; 5 Laboratory Animal Science Program, Leidos Biomedical Research, Frederick National Laboratory for Cancer Research sponsored by the National Cancer Institute, Frederick, Maryland, United States of America; University of North Carolina at Chapel Hill, UNITED STATES

## Abstract

Human gammaherpesviruses are associated with malignancies in HIV infected individuals; in macaques used in non-human primate models of HIV infection, gammaherpesvirus infections also occur. Limited data on prevalence and tumorigenicity of macaque gammaherpesviruses, mostly cross-sectional analyses of small series, are available. We comprehensively examine all three-rhesus macaque gammaherpesviruses -Rhesus rhadinovirus (RRV), Rhesus Lymphocryptovirus (RLCV) and Retroperitoneal Fibromatosis Herpesvirus (RFHV) in macaques experimentally infected with Simian Immunodeficiency Virus or Simian Human Immunodeficiency Virus (SIV/SHIV) in studies spanning 15 years at the AIDS and Cancer Virus Program of the Frederick National Laboratory for Cancer Research. We evaluated 18 animals with malignancies (16 lymphomas, one fibrosarcoma and one carcinoma) and 32 controls. We developed real time quantitative PCR assays for each gammaherpesvirus DNA viral load (VL) in malignant and non-tumor tissues; we also characterized the tumors using immunohistochemistry and *in situ* hybridization. Furthermore, we retrospectively quantified gammaherpesvirus DNA VL and SIV/SHIV RNA VL in longitudinally-collected PBMCs and plasma, respectively. One or more gammaherpesviruses were detected in 17 tumors; generally, one was predominant, and the relevant DNA VL in the tumor was very high compared to surrounding tissues. RLCV was predominant in tumors resembling diffuse large B cell lymphomas; in a Burkitt-like lymphoma, RRV was predominant; and in the fibrosarcoma, RFHV was predominant. Median RRV and RLCV PBMC DNA VL were significantly higher in cases than controls; SIV/SHIV VL and RLCV VL were independently associated with cancer. Local regressions showed that longitudinal VL patterns in cases and controls, from SIV infection to necropsy, differed for each gammaherpesvirus: while RFHV VL increased only slightly in all animals, RLCV and RRV VL increased significantly and continued to increase steeply in cases; in controls, VL flattened. In conclusion, the data suggest that gammaherpesviruses may play a significant role in tumorogenesis in macaques infected with immunodeficiency viruses.

## Author summary

Human gammaherpesviruses cause malignancies in HIV infected persons; in SIV infected macaques, gammaherpesvirus infections also occur. To understand the potential role of the rhesus gammaherpesviruses, RRV, RFHV and RLCV in cancers occurring in monkeys during SIV and SHIV studies spanning the last 15 years, we developed assays to measure the DNA viral load (VL) of each virus in these tumors and unaffected macaque tissues. We further characterized the tumors using immunohistochemistry and in situ hybridization, and quantified gammaherpesvirus DNA VL in PBMCs collected longitudinally during the original studies. We examined 18 animals with tumors and 32 controls. In all tumors, we detected one or more gammaherpesviruses; generally, one virus was predominant and very abundant compared to surrounding non-tumor tissues. SIV RNA VL in plasma and RLCV VL in PBMCs were independently associated with cancer risk. The longitudinal patterns of gammaherpesviruses VL, from SIV infection to death differed in cases and controls: while RFHV VL increased only slightly in all animals, RLCV VL and RRV VL increased significantly and continued to increase in cases, but flattened in controls. These data suggest that gammaherpesviruses may play a significant role in tumorogenesis in macaques infected with immunodeficiency viruses.

## Introduction

The lymphotropic human gammaherpesviruses Epstein Barr Virus (EBV) and Kaposi’s Sarcoma-Associated Herpesvirus (KSHV) are associated with malignancies in the setting of HIV infection[1]. EBV is associated with aggressive B cell Non-Hodgkin’s lymphomas such as diffuse large B cell lymphoma (DLBCL) and Burkitt’s lymphoma (BL), as well as classical Hodgkin’s lymphoma and other lymphoproliferative disorders. KSHV is associated with Kaposi’s sarcoma (KS), primary effusion lymphoma (PEL) and multicentric Castleman’s disease (MCD). While the incidence of AIDS-defining malignancies has declined since the introduction of potent anti-retroviral therapies, they still remain an important cause of morbidity and mortality in HIV infected persons [2, 3] and the risk of developing non-AIDS defining lymphomas and other malignancies is 2–3 fold higher in HIV infected individuals than in the general population [4, 5].

Rhesus macaques (*Macaca mulatta*) are the monkey species most widely-used in non-human primate (NHP) models of HIV infection; AIDS research conducted in this NHP species has provided many important insights into HIV pathogenesis and *in vivo* proofs of concept in the evaluation of preventive and therapeutic approaches (reviewed in: [6–8]). Three gammaherpesviruses are known to infect rhesus macaques: rhesus lymphocryptovirus (RLCV), closely related to EBV; and two rhadinoviruses, retroperitoneal fibromatosis herpesvirus (RFHV), closely related to KSHV and rhesus rhadinovirus (RRV). Experimental infection of macaques with RLCV and RRV have been used as models for AIDS-associated malignancies caused by gammaherpesviruses [9–13]. In addition, a related virus isolated from pig tailed macaques with lymphoma was shown to cause lymphoma in rabbits[14, 15]. However, the role of naturally occurring gammaherpesvirus infections in macaques subsequently infected experimentally with SIV/SHIV and their role in malignancies has not been extensively studied. Initial investigations at the Tulane National Primate Research Center reported on the prevalence of RLCV and RRV, but not RFHV, in healthy and SIV-infected macaques and suggested a role for RLCV, but not RRV, in SIV-associated lymphomas [16, 17]. A prevalence study of RRV and RFHV, but not RLCV, in healthy rhesus macaques in the California National Primate Research Center breeding colony has also been reported [18]. More recently, a retrospective study of RLCV, RRV and RFHV detection by PCR in lymphoma tumor samples from the Washington National Primate Research Center suggested a role for RLCV in SIV/SHIV related B cell lymphomas and RRV in SRV2-related T cell lymphomas, but RFHV was not detected. The study reported cross sectional PCR detection in tumor tissues only and did not examine non-tumor tissues or PBMCs [19].

We sought to further elucidate the potential role of naturally occurring gammaherpesvirus infections in malignancies arising in rhesus macaques experimentally infected with SIV/SHIV, by testing for gammaherpesvirus DNA samples from animals employed in SIV and SHIV studies conducted by the AIDS and Cancer Virus Program between 2001 and 2015, and comparing results from animals that either were or were not diagnosed with malignancies. First, we developed sensitive and specific real time quantitative PCR assays for RRV, RFHV and RLCV and used these to quantify DNA viral load (VL) in tumor tissue and adjacent unaffected tissues collected at necropsy from rhesus macaques that developed histologically confirmed malignancies. We then used immunohistochemistry and *in situ* hybridization to further characterize the malignancies occurring in these animals and their association with specific gammaherpesviruses. Finally, we determined the seroprevalence of all three gammaherpesviruses in the study animals and quantified gammaherpesvirus DNA VL in longitudinally collected PBMCs of the animals that had developed malignancies as well as SIV/SHIV infected control animals.

## Results

### Characteristics of the animals

Characteristics of the 18 cases and 32 control study animals are shown in Table 1. Controls were selected amongst animals euthanized for end-stage SIV/SHIV disease, whilst animals with tumors presented generally with symptomatic lesions that rendered euthanasia necessary, and that were histopathologically demonstrated to be malignant. Overall, cases and controls did not significantly differ at euthanasia in age, SIV/SHIV viral load or CD4 counts, and they were followed over a similar follow up period, although the cases were sampled more frequently; among the cases there were more females, which were significantly older (median: 5, interquartile intervals [IQR] 5–8 for male cases, median 16, IQR 11–16 for female cases).

**Table 1:** Characteristics of the study animals. Median and IQR are reported as applicable. Some data were unavailable for some animals.

	Cases	Controls	p
N	18	32	
Male sex	65%	91%	0.02
Duration of γ–HV VL follow up (days)	365(222–791)	377(288–702)	0.84
N longitudinal γ–HV VL assays	8 (6–11)	5(4–7)	0.001
*Characteristics at euthanasia*			
Age	6 (5–10)	5 (5–8)	0.24
CD4 + counts	190 (142–311)	358 (110–1034)	0.44
SIV/SHIV viral load/ mL	6.6x10^5^ (8.1x10^4^-6.9x10^6^)	6.6x10^5^ (1.2x10^5^-6.3x10^6^)	0.89
*Overall virological characteristics*			
γ–HV seropositivity			
RRV	94%	90%	0.63
RFHV	28%	25%	0.82
RLCV	81%	90%	0.38
median γ–HV viral load in PBMCs*			
RRV	12.5(0–157)	1 (0–16)	0.004
RFHV	0 (0–3)	0 (0–0)	0.88
RLCV	44 (3–208)	45 (7–141)	0.024

γ–HV, gammaherpesviruses, viral load calculated as copies/10^4^ PBMCs.

### Serology

Summary results of serological analyses are shown in Table 1. The prevalence of antibodies against each of the three viruses was similar in cases and controls. Antibodies against RRV and RLCV were detected in 94% and 81% of cases, respectively and in 90% of controls. Prevalence of antibodies against RFHV was lower, 28% in cases and 25% in controls. The prevalence of double and triple infection is shown in S2 Fig.

### Detection and quantification of viral DNA in tumor tissues

Formalin-fixed, paraffin-embedded (FFPE) blocks were available from eight macaques, six with lymphoma, one with fibrosarcoma and one with adenocarcinoma. Snap-frozen tumor specimen and frozen lymphocyte pellets from biopsies of lymphoid organs were available from an additional six and four macaques with lymphoma, respectively, for which FFPE had not been prepared. Multiple samples from each tumor and from surrounding unaffected tissues were examined. FFPE samples of four monkeys were microdissected by laser-capture or manually to obtain affected and adjacent unaffected tissues; in 11 other cases, adjacent unaffected tissue was obtained by separate sampling and identified histologically. For one animal with lymphoma no tissue specimen was available. Viral load data are summarized in Table 2 and in S1 Fig.

**Table 2:** Characteristics of the tumors.

Animal ID	Diagnosis	Site*		Tissue VL (copies /10,000 cells)*		
			Sample*	RRV	RFHV	RLCV
95D163	DLBCL	Jejunum	FFPE	6.7x10^2^	0	1.4x10^7^
A68Z	DLBCL	Maxilla	FFPE	†QP	0	2.4x10^7^
P436	DLBCL	Orbit, nose	SF	QP	0.6x10^1^	7.x10^4^
P787	DLBCL	Jejunum/LN	FL	6.7x10^2^	8x10^1^	7.2x10^5^
P857	DLBCL	Colon/LN	SF	2.7x10^1^	0	2.44x10^5^
18K	DLBCL	Jejunum/ LN	FFPE	QP	0	2.4x10^5^
95D191	DLBCL	Caecum/uterus	FFPE	0	0	1.3x10^5^
DBK7	DLBCL	Mesentery/LN/BM	FFPE	1.6x10^3^	QP	1.8x10^4^
DCBT	DLBCL	Caecum/Genito-urinary/LN	SF	QP	0	8.1x10^5^
P116	DLBCL	Mesentery, spleen/LN	SF	0.3x10^1^	QP	7.1x10^5^
XCC	DLBCL	Multi-organ	FFPE	0.4x10^1^	1.2x10^2^	1.3x10^6^
ZA43	DLBCL	Larynx, lung	SF	3x10^1^	0	2.4x10^5^
ZG17	DLBCL	Liver /nose/LN	SF	7.6x10^2^	0	7.1x10^5^
ZK37	DLBCL	Thymus	SF	QP	0	1.03x10^5^
DBWL	BL-like	Thyroid/pituitary/spleen/stomach/LN	SF	2.8x10^7^	0	1.2x10^2^
15J	BCL-U	Bladder/LN	ND	ND	ND	ND
AH49	Fibrosarcoma	Colon-rectum	FFPE	4.9x10^1^	8.6x10^5^	0
449Z	Carcinoma	Colon	FFPE	QP	0	QP

*In case of multiple sampling of tumor sites, the highest VL is reported. DLBCL, Diffuse large B-cell lymphomas; BL, Burkitt’st lymphoma, BCL-U, B cell lymphoma-unclassified. FFPE, fresh frozen paraffin embedded; SF, snap frozen; FL, frozen lymphocytes from a lymphoid organ biopsy. LN, lymph node; BM, bone marrow; GU genitourinary tract.

^†^QP, qualitative positive. Samples amplifying above assay threshold in one or more repeats and quantified as between 1 and 3 viral copies per reaction are classified as QP.

ND, not determined. For animal 15J, no tissue was available for testing.

Gammaherpesvirus DNA was detected by quantitative PCR in tumor tissues of four (RFHV), 14 (RRV) and 16 (RLCV) macaques. In 11 tumors, DNA from two of the viruses was detected while in three animals, tumors DNA from all three viruses was detected. Most lymphomas appeared to be RLCV-related based on the observed high RLCV viral loads and low or absent RRV and RFHV VL in tumor tissues. One animal, with a Burkitt-like lymphoma (BL) had extremely high RRV load, low RLCV and undetectable RFHV in the tumor tissue, which suggests a potential RRV etiology. Similarly, the animal with fibrosarcoma had a high RFHV viral load, very low RRV and undetectable RLCV in the tumor indicating a possible role for RFHV in this case. This is consistent with the known association between RFHV and retroperitoneal fibromatosis, a multifocal fibroproliferative syndrome that arises in the peritoneum, ileocecal junction and adjacent mesenteric lymph nodes in macaques with SIV/SHIV or SRV-2 infection[20]. The only animal with a carcinoma had low or undetectable viral loads for all three viruses; it is unlikely that gammaherpesviruses contributed to tumorigenesis in this case.

Gammaherpesvirus DNA was also detected in adjacent unaffected tissues, especially in lymph nodes, but levels were generally much higher in malignant tissues. In DLBCL cases, the median RLCV VL and interquartile range (IQR) were 4.0 x 10^2^ (12–1.3 x 10^3^) in tumors and 32 (0–1.3 x 10^5^) in unaffected tissues, p = 0.02; in the fibrosarcoma case, median RFHV VL was 7.2 x 10^5^ (IQR: 6.7 x 10^5^−8.5 x 10^5^) in tumor tissue and 1 (IQR: 0–1,1 x 10^3^) in unaffected tissues, p = 0.008; in the BL-like case, median RRV VL was 6.8 x 10^6^ (IQR: 1.4 x 10^6^−9.2 x 10^6^) in the tumor and 3.4 x 10^4^ (IQR: 2.4 x 10^4^−9.4 x 10^4^) in unaffected tissues, p = 0.003. Multiple samples from each tumor and from surrounding unaffected tissues were examined.

### Tumor immunophenotyping and gammaherpesvirus detection patterns

**Fig 1:**
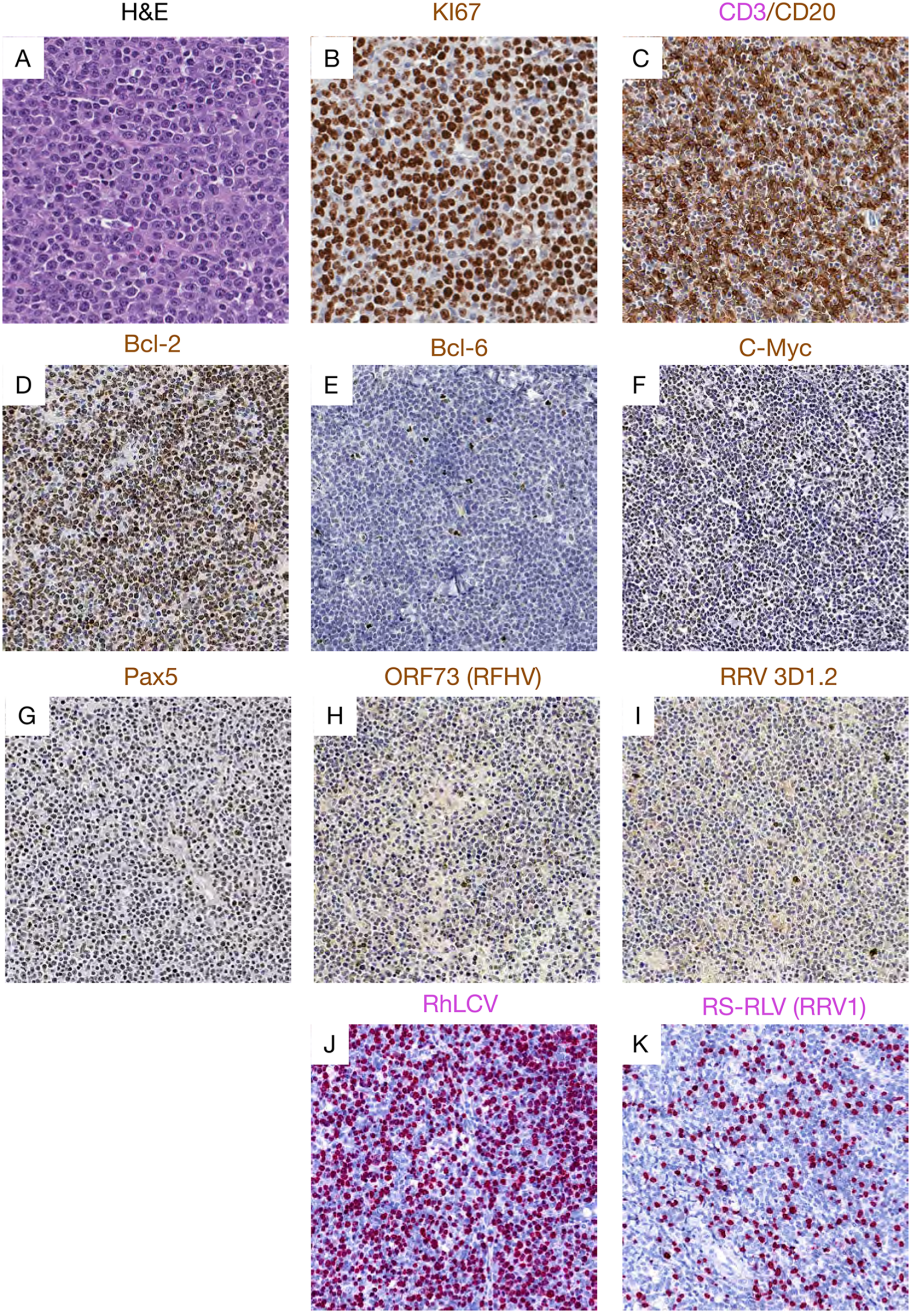
Representative pictures showing histopathology, immunohistochemistry and RNAscope staining representative of DLBCL cases. (A) H&E image showing diffusely distributed round or ovoid large lymphoid cells cells with vesicular nuclear chromatin, distinct central nucleoli, and scattered mitotic figures. Immunophenotyping showed (B) highly proliferative cells with strong Ki-67 expression, (C) CD20 expression, with admixed CD20-negative T-cells, (D) High expression of Bcl-2, (E) low expression of Bcl-6, (F) high expression of c-Myc, and (G) Pax5, but (H) low expression of the ORF73 protein from RFHV and (I) rare expression of RRV capside protein (clone 3D1.2). RNAscope showed (K) robust RhLCV viral RNA expression but (J)low RRV viral RNA expression in DLBCL cases.

**Fig 2:**
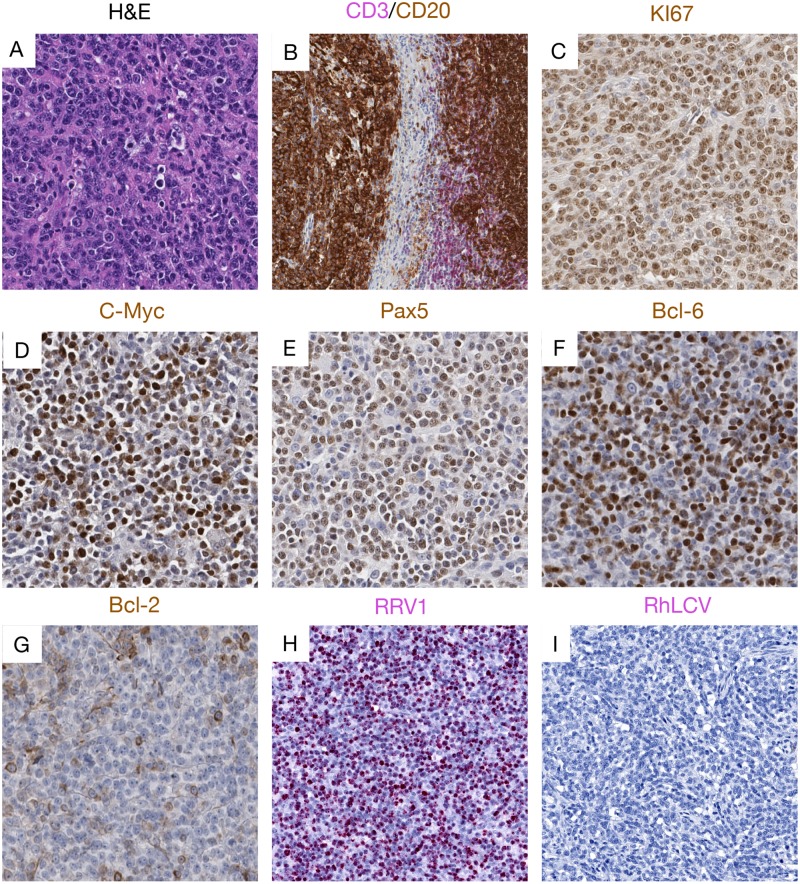
Representative pictures showing histopathology, immunohistochemistry and RNAscope staining of the BL-like lymphoma case. (A) H&E images showing atypical medium-sized lymphoid cells with clumped chromatin, with admixed small lymphocytes. Immunophenotyping showed (B) strong CD20 B cell expression and rare CD3+ T cells at the periphery of the tumor, (C) highly proliferative activity with strong Ki-67 expression, (D) c-Myc, (E) Pax- 5 and (F) Bcl-6 are found highly expressed, but with (G) lower Bcl-2 expression. RNAscope demonstrated (H) robust RRV viral RNA expression, but (I) rare RhLCV viral RNA expression in this case of BL-like lymphoma.

**Fig 3:**
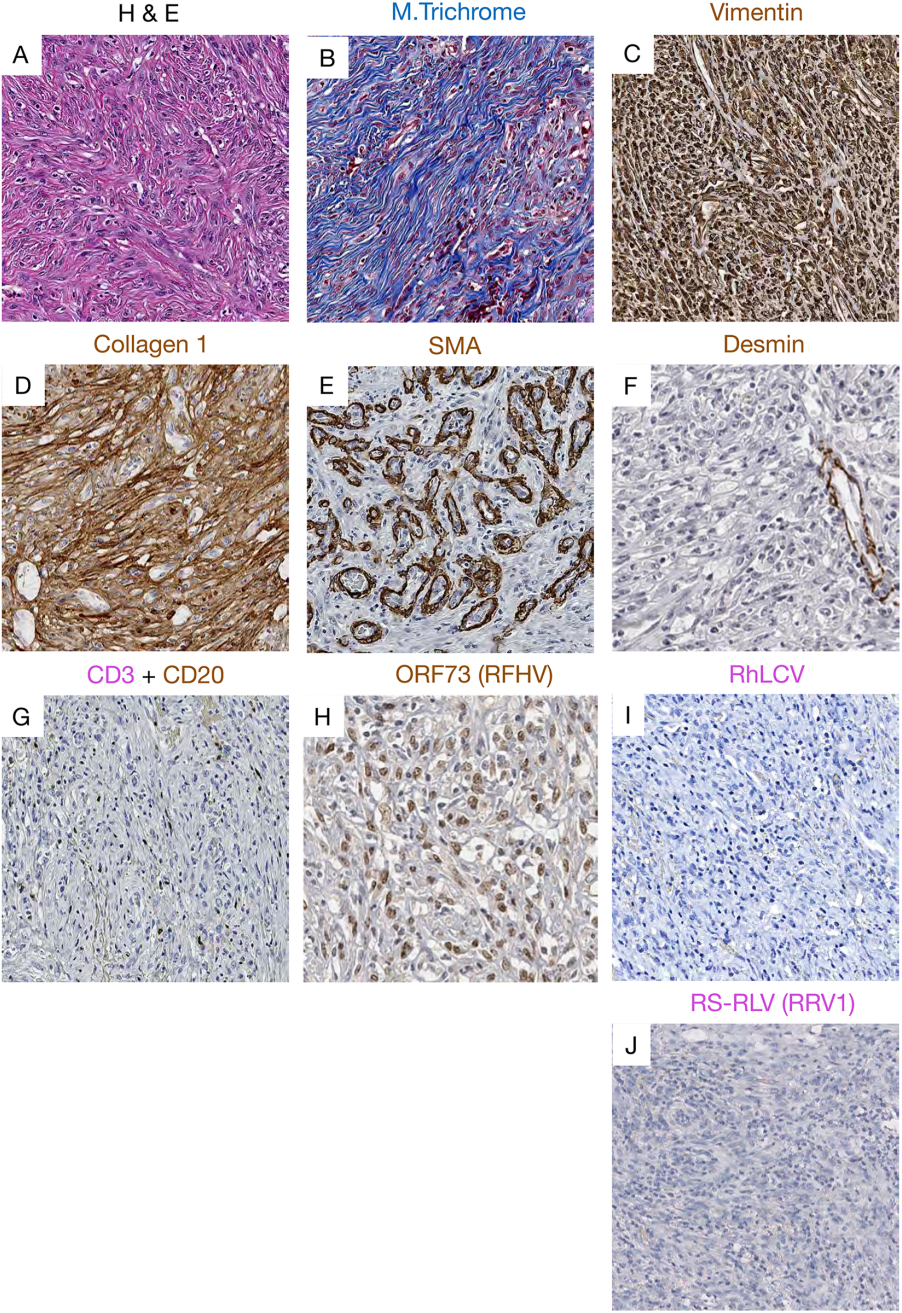
Representative pictures showing histopathology, immunohistochemistry and RNAscope staining of the fibrosarcoma case. (A) H&E image showing the elongated spindle cells with euchromatin and clear nucleoli. (B) Masson’s trichrome staining showing blue collagen staining within the fibrosarcoma. Immunophenotyping demonstrated strong expression of (C) vimentin, (D) collagen I within the fibrosarcoma. (E) SMA was highly expressed in vascular smooth muscle demonstrating high vascularization of the tumor, expression of (F) desmin restricted to vascular muscles, and (G) rare expression of CD20+ B cells marker. Using IHC we detected a strong nuclear signal for (H) ORF73 proteins attesting of RFHV infection. Using next generation RNAscope approach we were not able to detect any vRNA expression of (I) RLCV or (J) RRV.

We performed detailed immunohistochemistry using a panel of antibodies specific for Bcl-2, Bcl-6, CD3, CD20, c-Myc, Ki-67 and Pax5. Histologically, 14 lymphomas were classified as diffuse large B-cell lymphoma (DLBCL). One tumor was categorized as BL—like and one tumor had features of both DLBCL and BL, and was characterized pathologically as a B cell lymphoma, unclassified (BCL-U)[21, 22]. Of the three remaining malignancies, one was a fibrosarcoma, one a carcinoma and one, histopathologically classified as lymphoma at necropsy, had no tissue available for further analyses. S4 Table shows the sites, gross exam, histologic and *in situ* hybridization findings made in all the tumors tested. A representative DLBCL case, and the single BL-like and fibrosarcoma cases are shown in Figs 1–3.

Lymphoma tissues consisted of different populations of lymphocytes, centroblasts, and immunoblasts, many with plasmacytoid differentiation with heterogenous expression of both CD20 and Pax5, showing tumor cells at different stages of differentiation. This was also evident from the variable presence of euchromatin, prominent nucleoli, and frequency of mitotic figures. All DLBCLs were strongly positive for Ki-67, c-Myc, and Bcl-2 but negative for Bcl-6 (Fig 1) while the BL-like case was positive for Bcl-6 but low for Bcl-2 [23](Fig 2).

The tumor diagnosed as colonic fibrosarcoma was composed of elongated spindle cells with infiltration of many neutrophils (Fig 3). The neoplastic cells were negative for desmin but expressed abundant vimentin and collagen I. (Fig 3).

To further explore a potential direct role of gammaherpesvirus infection in these tumors, we performed immunohistochemistry on tissues using antibodies specific for the EBV LMP1 and EBNA1, that have been demonstrated to be cross-reactive with the RLCV orthologs, as well as anti-RRV major capsid protein (clone 3D1.2), and anti-KSHV ORF73, with known cross-reactivity to the RFHV ortholog [24]. In addition, we performed RNAscope *in situ* hybridization using probes designed to specifically target RNA from RLCV, RRV (Figs 1–3) and RFHV. High RLCV RNA expression was seen in all DLBCLs but not in the BL-like case (Figs 1 and 2). Interestingly, cells with low levels of cytoplasmic RLCV LMP1 were seen in both non-malignant and B-cell lymphoma (BCL) tissues, however, few cells with RLCV EBNA1 were found in the non-malignant tissues. In addition, only rare RRV positive cells were seen in DLBCL tissues (Fig 1). In stark contrast to DLBCL cases, elevated levels of RRV and no RLCV were seen in the one BL-like case (Fig 2). In the fibrosarcoma, only tumor cells were positive for KSHV ORF73, while all other BCL and non-malignant tissues were negative or low (Fig 3). In the fibrosarcoma tumor case, we were not able to detect RRV RNA or protein (Fig 3). Collectively, these findings are in line with our tissue PCR results and strongly suggest association of RLCV with DLBCLs, RRV with a BL-like tumor, and RFHV with a KS-like tumor in NHPs.

### Detection and quantification of viral DNA in PBMCs

Median RRV and RLCV DNA viral load were statistically significantly higher in PBMCs of cases than controls. RFHV was detectable in PBMCs of few animals and viral load did not differ between cases and controls (Table 1). In a multivariate longitudinal analysis including age at necropsy, sex, SIV/SHIV plasma VL, CD4 counts, and gammaherpesvirus PBMC viral loads, SIV/SHIV plasma VL and RLCV DNA viral load in PBMCs were independently associated with odds of developing cancer but RRV and RFHV DNA viral loads in PBMCs were not. (Table 3).

**Table 3:** Multivariable longitudinal analysis odds of cancer diagnosis as a function of age, sex, CD4+ counts, SIV and gammaherpesvirus viral loads.

	OR	95%	CI	p
Plasma SIV/SHIV	4.44	2.21	8.92	**<0.001**
PBMC RRV	1.76	0.68	4.55	0.241
PBMC RFHV	2.78	0.73	10.64	0.135
PBMC RLCV	5.73	1.92	17.11	**0.002**

SIV/SHIV Log10 copies/mL, gammaherpesviruses Log10 copies/^104^ cells

**Fig 4:**
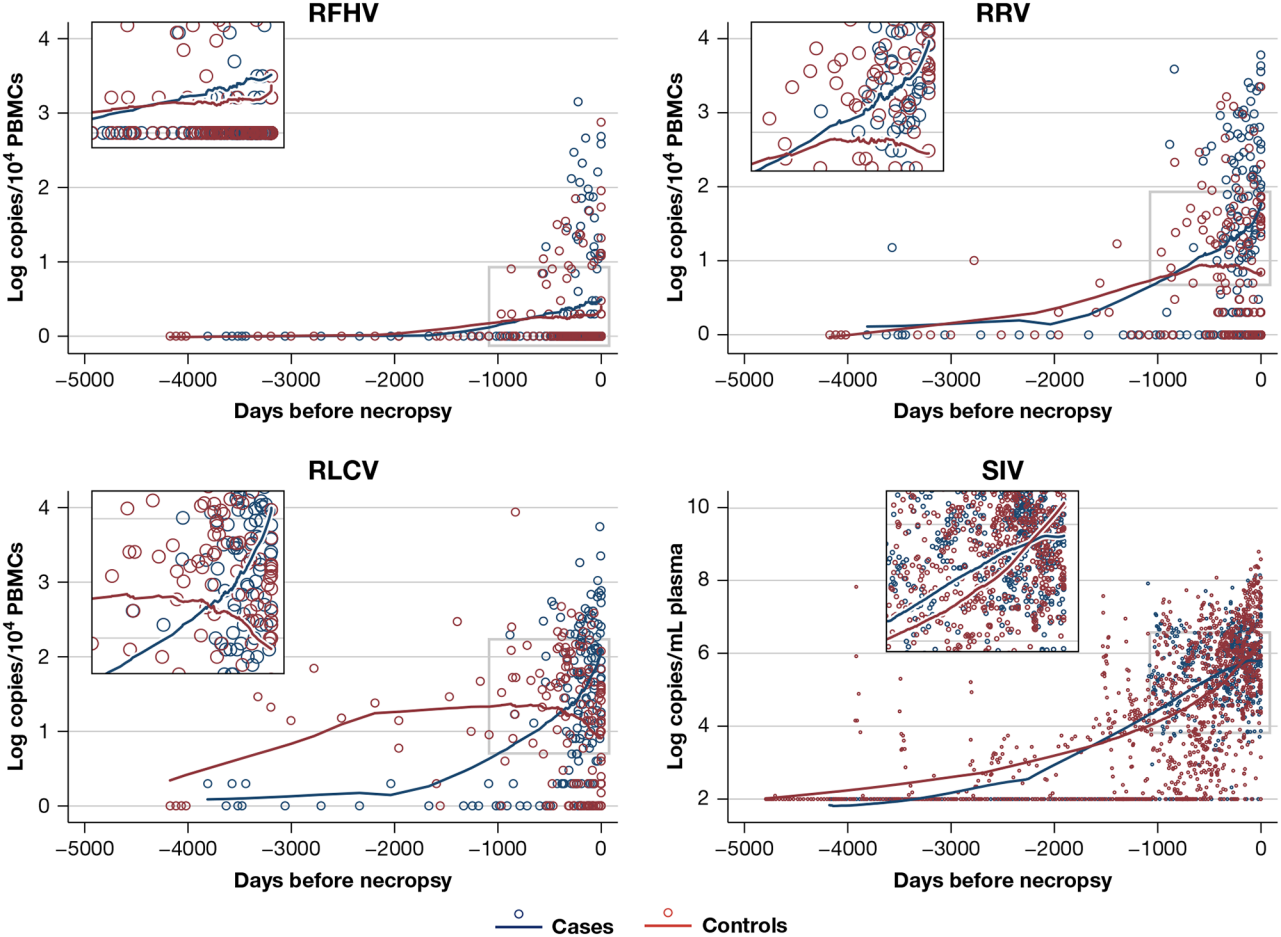
Longitudinal trend of SIV/SHIV viral load in plasma and gammaherpesvirus viral loads in PBMCs after SIV/SHIV infection. Circles indicate actual measurements, lines represent LOWESS (LOcally WEighted Scatterplot Smoothing). VL, viral load, log10 copies per 10^4^ cells for gammaherpesviruses, log copies/mL for SIV/SHIV.

Univariate non-parametric local regressions showed that the longitudinal pattern of VL levels in the PBMCs of cases and controls differed for each virus (Fig 4). For RFHV, PBMC DNA viral load increased only slightly in both cases and controls from SIV infection to necropsy. For RLCV and RRV DNA, viral loads increased significantly in both groups upon SIV infection and continued to increase steeply in cases until diagnosis and necropsy, whereas in controls, the VL flattened. This pattern is consistent with the detection of high levels of RLCV in most of the malignant tissues in the study while the pattern seen for RRV VL is more unexpected since only one animal had an RRV VL in malignant tissue.

## Discussion

Infections with lymphotropic gammaherpesviruses are prevalent in rhesus macaques used in HIV research and yet are rarely evaluated, except in limited specific pathogen free colonies stringently bred, tested, and cared for to exclude these agents. In our retrospective study, antibodies to RLCV and RRV were present in >90% of cases and controls, while antibodies to RFHV were detected in 25% of the cases and 28% of the controls. These serological data are similar to previous reports from US primate centers [16–18]. Our objectives for this study, which was more extensive than most prior published surveys, and included longitudinal quantitative PCR analysis for all three rhesus herpesviruses as well as histologic (IHC/ISH) analysis comparing tumor tissue and corresponding non-tumor tissue, were to elucidate the potential role of naturally acquired gammaherpesviruses in malignancies occurring in SIV/SHIV infected macaques. We have found that most such malignancies were associated with RLCV, consistent with previous reports. [16, 17, 19]. We did observe however, a BL-like tumor with a very high RRV viral load, low or absent RFHV and RLCV, and IHC staining consistent with a RRV etiology. We also observed a fibrosarcoma in which immunohistochemistry and viral load in the tumor were consistent with an etiological role for RFHV. Thus, we show that all three gammaherpesviruses may have oncogenic potential in the setting of experimental SIV/SHIV infection. The histological classification of these malignancies observed in SIV/SHIV-infected macaques broadly resembles that of malignancies seen in HIV-infected persons.

Retrospective longitudinal analyses of gammaherpesvirus load in PBMC samples from animals that did develop lymphomas showed a marked increase of RLCV DNA viral load in cases from SIV/SHIV infection to diagnosis, whereas in controls it eventually decreased, consistent with the association between RLCV reactivation and lymphoma. All the malignancies, except the carcinoma, were diagnosed at or shortly before necropsy, therefore it is unclear from our data whether elevated levels of RLCV preceded the development of lymphoma or accompanied it. Prospective analyses conducted in future NHP studies will be needed to further elucidate this dynamic. Interestingly, whilst not an independent risk factor, RRV viral load also increased over time from SIV/SHIV infection in all animals developing lymphoma, whereas it eventually decreased in controls, even though, among the more limited subset of animals seropositive for this gammaherpesvirus, only one animal developed a lymphoma showing exclusively RRV in tumor tissue. On the contrary, in infected animals, RFHV DNA VL did not show differential kinetics between cases and controls. Further studies in additional animals will be necessary to understand the relationship between RRV replication and the pathogenesis of lymphomas associated with RLCV. Besides RLCV DNA VL, high SIV/SHIV RNA VL was independently associated with cancer risk, emphasizing the role of progressive SIV/SHIV infection and accompanying immunodeficiency.

While provocative, the retrospective, correlative nature of these data does not permit the direct demonstration of a causative etiologic role for these viruses in the observed malignancies. Further, the retrospective nature of the results and limited samples available from studies that were conducted with other primary objectives do not allow us to directly address the role of progressive SIV/SHIV virus associated immunodeficiency and, potentially, declines in specific immune responses to the gammaherpesviruses in contributing to development of tumors. Similarly, the potential role of an immunoinflammatory milieu engendered by uncontrolled AIDS virus replication and associated host responses in breaking gammaherpesvirus latency remains unaddressed by the present results. However, our observations extend previous work in important ways by providing longitudinal and comparative data for all three rhesus gammaherpeseviruses in the same animals, along with detailed comparative analysis using serological assays and quantitative PCR, as well as immunohistochemical and *in situ* hybridization analyses of both tumor and control tissues.

While falling short of a “smoking gun”, in aggregate, the results strongly implicate rhesus gammaherpesviruses in contributing to the development of tumors in the setting of experimental SIV/SHIV infection and point to the importance of careful further studies to address questions of etiologic roles and mechanisms of pathogenesis. Our study suggests that RLCV and RRV are likely to play a significant role in lymphomagenesis in SIV/SHIV infected macaques and that the contribution of gammaherpesviruses to SIV-associated malignant disease is worthy of further study, particularly as these tumors recapitulate many important features of malignancies that continue to arise in HIV-infected humans, malignancies for which an animal model to evaluate novel treatment approaches would be valuable.

## Materials and methods

### Animal and sample selection

Specimens from eighteen animals with malignancies (16 with lymphoma, one with carcinoma and one with fibrosarcoma) were available from studies conducted with a variety of different primary objectives. All selected animals were rhesus macaques (*Macaca mulatta*) of Indian origin and were infected with SIV or chimeric simian-human immunodeficiency virus (SHIV). Malignancies were demonstrated at necropsy in all cases. Thirty-two control animals without malignancies were also selected from the same studies; for these animals, the indication for euthanasia was end-stage SIV/SHIV disease.

Plasma and peripheral blood mononuclear cells (PBMCs) were prepared from whole blood collected in EDTA anticoagulated Vacutainer tubes (BD). Plasma was separated from whole blood by centrifugation, recentrifuged to eliminate cells, platelets and debris, then aliquoted and then stored at -80°C until analysis. PBMCs were isolated from whole blood by Ficoll-Paque Plus (GE Healthcare) gradient centrifugation. Portions of isolated PBMCs were pelleted in a microcentrifuge and all liquid was removed prior to storage at -80°C. Plasma was tested for gammaherpesvirus serology at necropsy as described below. Stored PBMCs collected up to 4186 days prior to necropsy were available from a median of 11 time points (interquartile range [IQR}8–13) for cases and 6 time points for controls (IQR 5–12). Formalin fixed, paraffin embedded (FFPE) blocks or snap frozen/cryopreserved specimen of tumors and surrounding unaffected tissues were available for 17 of the cases.

### Serology testing

Antibodies to RRV were detected using peptide based ELISAs (ORF 65, R8.1 and ORF 73) as previously described [24]. Antibodies to RLCV were detected using peptide based ELISAs (VCA) as previously described [25], [26]. Antibodies to RFHV were detected based on ELISA cross reactivity to the recombinant ortholog KSHV proteins K8.1 and ORF 73, as previously described[27].

### DNA extraction

DNA from frozen tissue samples was extracted using Trizol according to the manufacturer’s instructions after tissue homogenization using a gentleMACS dissociator (Miltenyi Biotec). DNA from FFPE tissues was extracted using the phenol-based AutoGenprep 245T Animal Tissue DNA Extraction Kit (Autogen) according to the manufacturer’s method. DNA was extracted from PBMC pellets using QIAamp DNA blood mini kit (QIAGEN) according to the manufacturer’s instructions. Yield and purity were determined by NanoDrop 1000 spectrophotometer (NanoDrop Technologies). DNA was stored at -20°C until subsequent assays/analyses.

### QPCR development

To develop plasmids for assay standard curves, animals seropositive for each virus were identified and nested PCR was used to amplify DNA from corresponding PBMC samples with primers specific for each virus (Jumpstart ReadyMix, Sigma) using cloning primers listed in S1 Table. Cloning primers were designed using GeneRunner software (version 4.0.9.56 Beta) and synthesized by Eurofins-Operon. PCR products of the expected size were excised from gels and purified (QIAquick, Qiagen). Products were cloned into Promega’s T-Easy vector system II (Promega) and sequenced using a 3130XL Applied Biosystems sequence detection system (Thermo Scientific). For each virus, sequences were obtained from multiple seropositive animals and aligned with available reference sequences from GenBank. Consensus sequences were generated and used to design primers and probes for real-time quantitative PCR (qPCR) using Primer Express software version 3.0.1 (Applied Biosystems, Thermo Scientific). In the case of RRV, reported sequence variation of the glycoprotein B region was incorporated into the design [28]. Plasmids were purified and concentrated in a separate building by the Protein Expression Laboratory (Leidos Biomedical, Frederick National Laboratory for Cancer Research) to prevent template contamination of ACVP laboratory areas. All probes were labeled with FAM (reporter) and TAMRA (quencher). Primers and probes are listed in S1 Table. Plasmid stocks were quantitated by Nanodrop 1000 (Thermo Scientific) and 10-fold serial dilutions were made using 1 X TE buffer pH 7.0 (Ambion) with 0.1 μg/ml fish sperm DNA (Ambion) resulting in standard curve linear dynamic ranges from 10^6 to 10^0 copies. Three standard curve dilution series were made for each assay and tested in triplicate reactions across 10 individual plates to determine inter-assay variability as shown in S2 Table. Each assay was optimized using universal master mix (Applied Biosystems) with final primer/probe concentrations of 100 nM. The cycling conditions for all assays consisted of a 2-minute hold at 50°C, 95°C hold for 10 minutes followed by 45 cycles performed at 95°C for 15 seconds, 55°C for 30 seconds, and annealing at 60°C for 1 minute. SIV/SHIV RNA viral loads in plasma were determined by quantitative qRT-PCR as previously described[29], with progressively improved threshold sensitivity of the assay over the period covered by the study through refinements of the assay as described[30].

### Cell quantitation assay

Quantitative PCR for rhesus CCR5 was performed largely as previously described [31] in order to determine the number of cell-equivalents. The CCR5 probe was adapted with FAM/TAMRA modifications and ran as a single-plex qPCR assay on an Applied Biosystems 7900 HT sequence detection system. Inter-assay standard curve performance in shown in supplemental Table 2.

### Microdissected tissue-manual

Serial 7 μm sections were cut and individually placed onto positively charged glass microscope slides. Tumor and unaffected tissue regions, as annotated by a pathologist, were dissected without the removal of the paraffin. Using a single edge razor blade and a dissecting microscope, unwanted tissue was removed from slides and discarded. The unaffected tissue from all slides of one specimen were removed and placed into a single 1.5 ml microcentrifuge tube for DNA extraction. Then the tumor tissue from the same specimen was removed from all slides and placed into another 1.5 ml microcentrifuge tube.

### Microdissected tissue-laser capture

Laser capture microdissection (LCM) and collection of annotated targets from serial 7 μm FFPE sections was performed on a MMI CellCut Plus microdissection instrument (MMI Molecular Machines&Industries, Glattbrugg, Switzerland) with the following settings: laser speed-37%, laser focus-78% and laser power-41%. LCM workflow, LCM slide preparation, target dissection and collection were carried as previously described for FFPE NanoString LCM sample[32] DNA extraction was performed using the DNA extraction protocol used for hand-micro dissected samples.

### Cell line controls for immunohistochemistry

Cell line controls for IHC were developed using B cell lines: Ramos (virus negative); BCBL-1 (KSHV positive); Namalwa (EBV positive); LCL 8664 (RLCV positive); and BJAB-RRV (RRV positive). BJAB-RRV was a kind gift from Dr. Blossom Damania, UNC Chapel Hill. Approximately 10 million cells from each cell line were pelleted and the cell pellets were fixed in 4% PFA overnight at room temperature then wash in 80% ethanol, mixed with HistoGel (HG-400-012, Thermo Scientific) and stored at 4°C until processing for paraffin embedding. Infected and uninfected cell lines were embedded into one block for direct comparison.

### Immunohistochemistry and RNAscope *in situ* hybridization

FFPE sections were dewaxed in xylenes and rehydrated with serial washes of ethanol to water. Heat inducted epitope retrieval was performed in 0.01% citraconic anhydride buffer (pH 7.4) in a Decloaker pressure cooker (Biocare Medical, Inc.) programed for 30 seconds at 122°C. After slides were cooled to room temperature, rinsed for 5–10 min in ddH2O, slides were incubated for 30min in blocking buffer [1x TBS-Tween20 (0.05%) containing 0.25% casein protein] to block non-specific staining. After removing the blocking solution, the slides were incubated with primary antibodies diluted in blocking solution overnight at 4°C or RT. Slides were placed in wash buffer (1x TBS-Tween20 (0.05%)) for 5 min and endogenous hydrogen peroxidases were quenched by incubating slides in 1.5% hydrogen peroxide in TBS buffer for 10 min. Detection of gammaherpesvirus specific antibodies was performed using the mouse or rabbit Polink polymer staining system (Golden Bridge International, Inc) according to manufacturer’s instructions then Impact DAB (3,3′-diaminobenzidine; Vector Laboratories) as previously reported [33]. Slides were counterstained with hematoxylin and mounted with Permount. Details of primary antibodies and used dilutions are shown in S3 Table. Double staining for CD3 and CD20 was done with Warp Red (Biocare Medical, Inc.) as chromogen for CD3 (red) and DAB for CD20 (brown). Slides were scanned at high magnification (× 200) using a whole-slide scanning microscope (Aperio AT2 System, Aperio Technologies), yielding high-resolution data from the entire tissue section [33].

RNAscope *in situ* hybridization was performed as described previously [34]Briefly, following HIER (Pretreat 2 step; Advanced Cell Diagnostics, ACD) and proteinase digestion (Pretreat 3 step, ACD), the slides were incubated 2 hours at 40°C with either rLCV (ACD-ref:448011), RRV (ACD-ref:448021) or RFHV (ACD-ref:448031) probe. Amplification steps were performed according to the ACD protocol with the exception that all wash steps used a 0.5X wash buffer. Slides were scanned at high magnification (×400) using a whole-slide scanning microscope (Aperio AT2 System, Aperio Technologies), yielding high-resolution data from the entire tissue section.

### Statistical analysis

A Mann-Whitney test was used to compare mean log viral load and other continuous variables between cases and controls in univariate analyses, and equality of proportions was tested using large-sample statistics. Hierarchical linear models were used for comparing viral load in tumor and unaffected tissues across multiple animals. Univariate non-parametric regressions (LOWESS) and multivariate longitudinal random effects models were used for longitudinal analyses. All statistical analyses were performed using Stata v13.

### Ethics statement

This study made use of samples from Indian-origin rhesus macaques that were housed at the National Institutes of Health (NIH) and cared for in accordance with the Association for the Assessment and Accreditation of Laboratory Animal Care (AAALAC) standards in an AAALAC-accredited facility, and all procedures were performed according to protocols approved by the Institutional Animal Care and Use Committee of the National Cancer Institute (Assurance #A4149-01) and adhered to the standards of the NIH “Guide for the Care and Use of Laboratory Animals” (National Research Council. 2011. Guide for the care and use of laboratory animals, 8th ed. National Academies Press, Washington, DC). Twenty-six purpose-bred Indian-origin rhesus macaques (Macaca mulatta) weighing on average 7kg (range 5-9kg) were housed at the National Institutes of Health (NIH) and cared for in accordance with the Association for the Assessment and Accreditation of Laboratory Animal Care (AAALAC) standards in an AAALAC-accredited facility and all procedures were performed according to protocols approved by the Institutional Animal Care and Use Committee of the National Cancer Institute (Assurance #A4149-01). Animals were maintained in Animal Biosafety Level 2 housing with a 12:12-hour light: dark cycle, relative humidity 30% to 70%, temperature of 23 to 26°C and all animals were observed twice daily by the veterinary staff. Filtered drinking water was available ad libitum, and a standard commercially formulated nonhuman primate diet was provided thrice daily and supplemented 3–5 times weekly with fresh fruit and/or forage material as part of the environmental enrichment program.

Environmental enrichment: Each cage contained a perch, two portable enrichment toys, one hanging toy, and a rotation of additional items (including stainless steel rattles, mirrors, and challenger balls). Additionally, the animals were able to listen to radios during the light phase of their day and were provided with the opportunity to watch full-length movies at least three times weekly. At the start of the study, all animals were free of cercopithecine herpesvirus 1, simian immunodeficiency virus (SIV), simian type-D retrovirus, and simian T-lymphotropic virus type 1. All animals were treated with enrofloxacin (10 mg/kg once daily for 10 days), paromomycin (25 mg/kg twice daily for 10 days), and fenbendazole (50 mg/kg once daily for 5 days) followed by weekly fecal culture and parasite exams for 3 weeks to ensure they were free of common enteric pathogens. At least a 4-week post treatment period allowed time for stabilization of the microbiome prior to use in this study.

Physical Examination: All animals received complete physical examinations during preventative healthcare. Examinations were performed under anesthesia, generally using ketamine (10–25 mg/kg, IM), telazol (4–10 mg/kg, IM), and/or dexmedetomidine (7.5–15 μg/kg, IM), and generally concurrently with other procedures (e.g. phlebotomy) to reduce the total number of anesthesia events required. During all procedures, animals were monitored by vet technical staff. All monkeys were observed at least twice daily by trained veterinary technical staff for any abnormal signs or behaviors. Supportive treatment was administered as suggested by the clinical veterinarian.

Animal Procedures: All procedures were performed using chemical restraint unless specifically mentioned otherwise to ensure the safety of both staff and animals. Choice of anesthetic depended on the procedure, but was generally performed using ketamine (10–25 mg/kg, IM), telazol (4–10 mg/kg, IM), and/or dexmedetomidine (7.5 15 g/kg, IM). All of these drugs are commonly used in nonhuman primates and are considered safe and effective.

For euthanasia, animals were initially sedated with ketamine (10–25 mg/kg, IM) or telazol (4–10 mg/kg, IM) followed by an overdose of sodium pentobarbital (>75 mg/kg, IV) to effect, in accordance with ACUC guidelines.

## Supporting information

S1 Fig Intratumoral viral load at necropsy. VL, viral load, log10 copies per 10^4^ cells. DLBCL, Diffuse large B cell lymphoma; BL, Burkitt lymphoma-like tumor; Sar, Fibrosarcoma; Ca, Carcinoma.
(TIF)

S2 Fig Number of double and triple gammaherpesvirus infections in cases and controls. (TIF)

S3 Fig Immunohistochemistry and ISH staining on positive and negative control cell lines LCL 8664 and Ramos, respectively, demonstrating cross reactivity of EBV antibodies and specificity of probes for RLCV. (TIF)

S1 Table Characteristics of PCR assays developed to detect rhesus gammaherpesviruses. RRV, rhesus rhadinovirus; RLCV, rhesus lymphocryptovirus; RFHV, rhesus retroperitoneal fibromatosis virus; gB, glycoprotein B; pol, polymerase. All probes were labeled with FAM (reporter) and TAMRA (quencher). Highlighted bases (bold) indicate areas of sequence variability.
(DOCX)

S2 Table Inter-Assay estimated copy variation for serial dilution of plasmid fragment, copy number from 10^1^ to 10^6^. The average copy number was calculated from ten PCR assays in triplicate.
(DOCX)

S3 Table Primary antibodies and working conditions used in IHC. (DOCX)

S4 Table Characterization of all tumours tested including sites, gross exam, histologic and *in situ* hybridization observations. (DOCX)
