# Supplementary figures and images for: Gammaherpesvirus infection and malignant disease in rhesus macaques experimentally infected with SIV or SHIV
Source: PLoS Pathog. 2018 Jul 12;14(7):e1007130. doi: 10.1371/journal.ppat.1007130 (PMC6042791; doi:10.1371/journal.ppat.1007130)

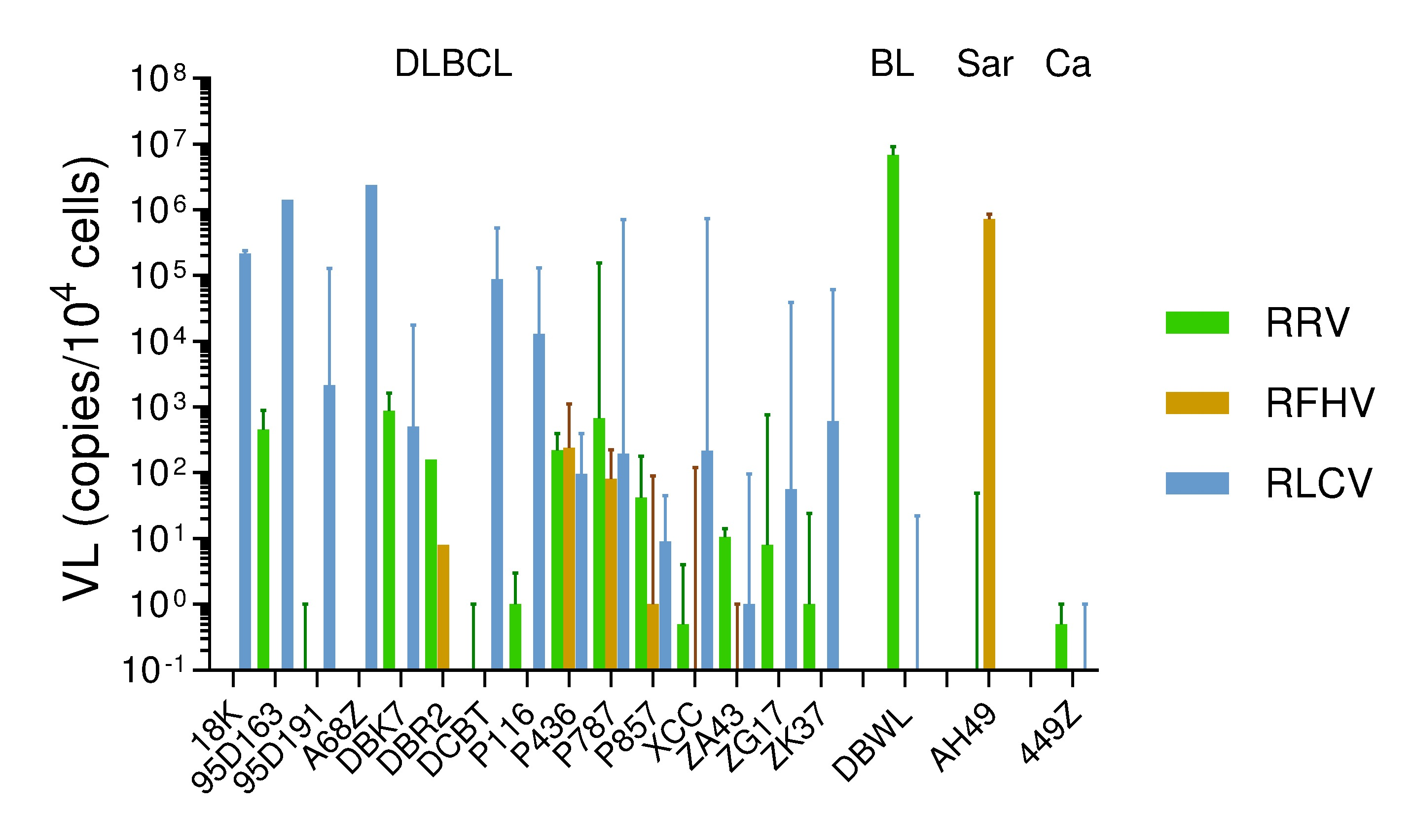

Supplement: S1 Fig — VL, viral load, log10 copies per 104 cells. DLBCL, Diffuse large B cell lymphoma; BL, Burkitt lymphoma-like tumor; Sar, Fibrosarcoma; Ca, Carcinoma. (TIF) [file ppat.1007130.s001.tif]

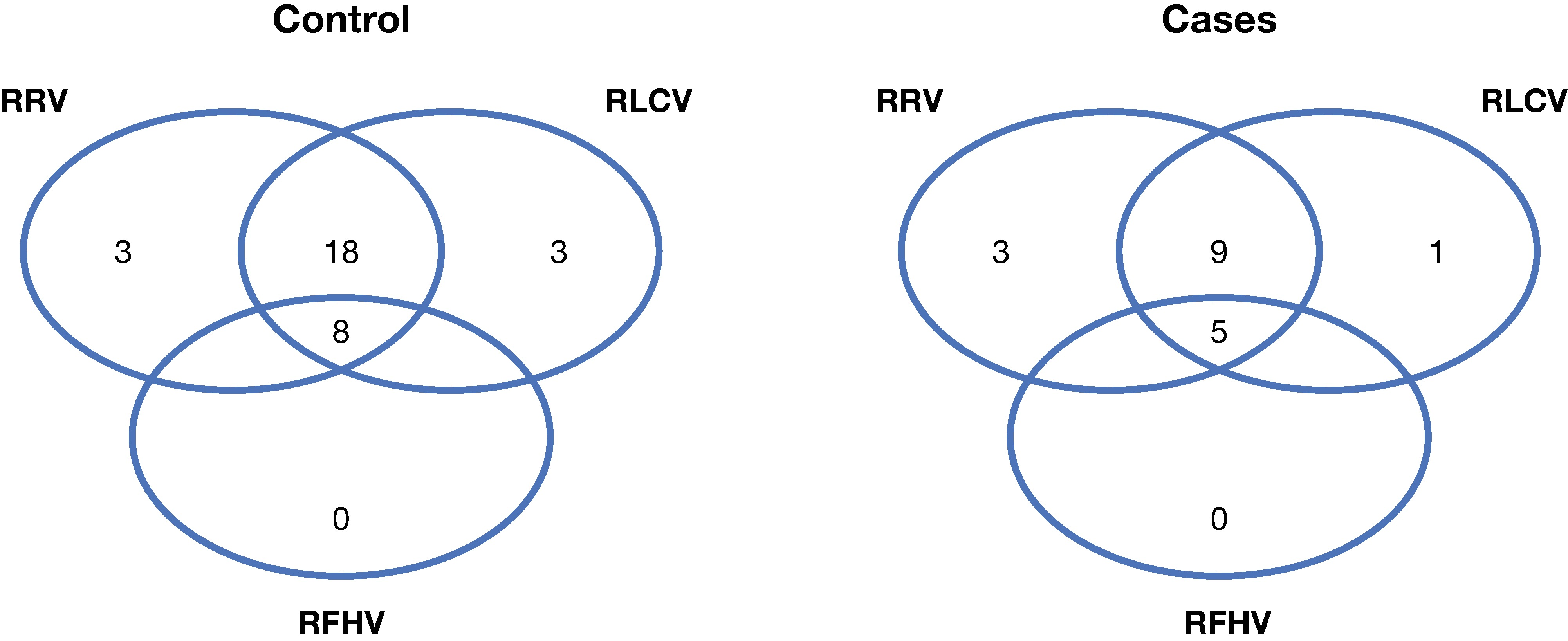

Supplement: S2 Fig — (TIF) [file ppat.1007130.s002.tif]

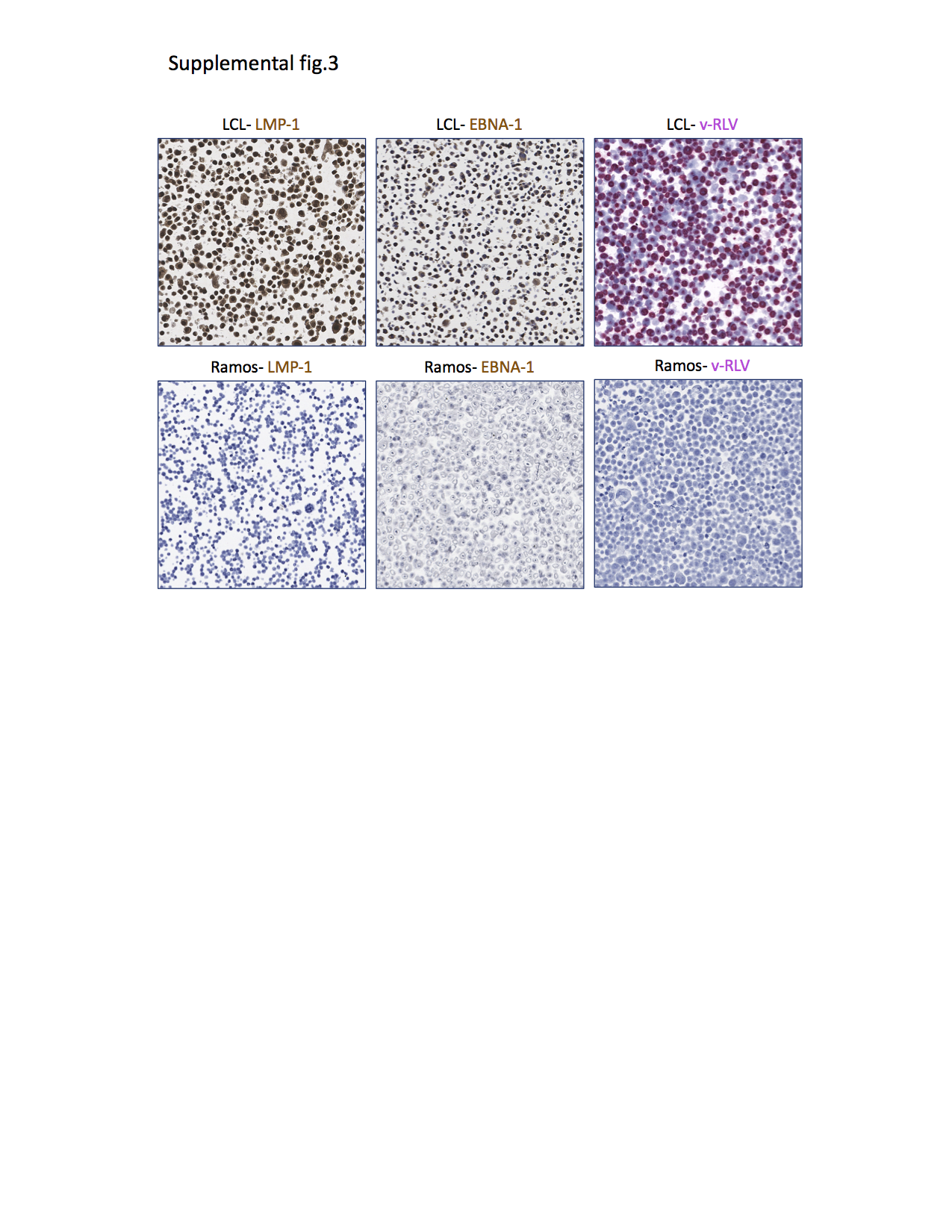

Supplement: S3 Fig — (TIF) [file ppat.1007130.s003.tif]

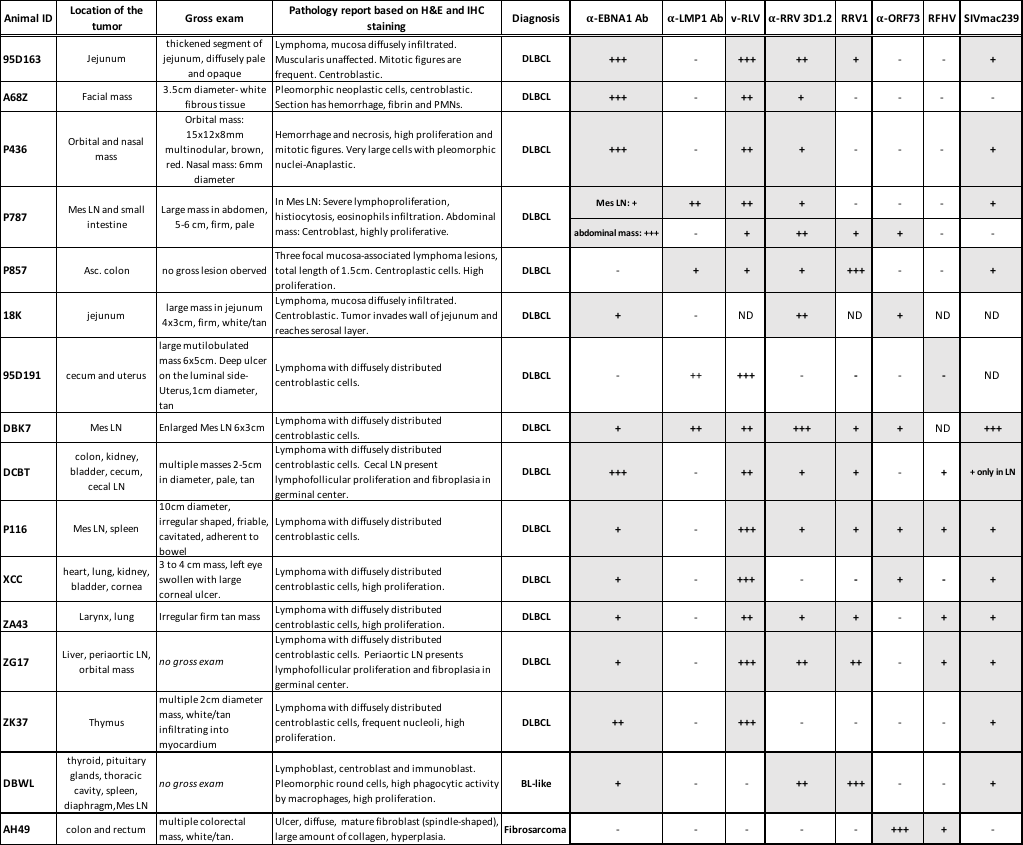

Supplement: S4 Table — (DOCX) [file ppat.1007130.s007.docx]
