# Supplementary material for: Gammaherpesvirus infection and malignant disease in rhesus macaques experimentally infected with SIV or SHIV
Source: PLoS Pathog. 2018 Jul 12;14(7):e1007130. doi: 10.1371/journal.ppat.1007130 (PMC6042791; doi:10.1371/journal.ppat.1007130)
Supplement: S1 Table — RRV, rhesus rhadinovirus; RLCV, rhesus lymphocryptovirus; RFHV, rhesus retroperitoneal fibromatosis virus; gB, glycoprotein B; pol, polymerase. All probes were labeled with FAM (reporter) and TAMRA (quencher). Highlighted bases (bold) indicate areas of sequence variability. (DOCX) [file ppat.1007130.s004.docx]

Table S1: Characteristics of PCR assays developed to detect rhesus gammaherpesviruses. RRV, rhesus rhadinovirus; RLCV, rhesus lymphocryptovirus; RFHV, rhesus retroperitoneal fibromatosis virus; gB, glycoprotein B; pol, polymerase. All probes were labeled with FAM (reporter) and TAMRA (quencher). Highlighted bases (bold) indicate areas of sequence variability.
