# Supplementary material for: Gammaherpesvirus infection and malignant disease in rhesus macaques experimentally infected with SIV or SHIV
Source: PLoS Pathog. 2018 Jul 12;14(7):e1007130. doi: 10.1371/journal.ppat.1007130 (PMC6042791; doi:10.1371/journal.ppat.1007130)
Supplement: S2 Table — The average copy number was calculated from ten PCR assays in triplicate. (DOCX) [file ppat.1007130.s005.docx]

Table S2. Inter-Assay estimated copy variation for serial dilution of plasmid fragment, copy number from 10^1^ to 10^6^. The average copy number was calculated from ten PCR assays in triplicate.

| Estimated copy number | **Measured copy number** | | | | | | | |
| --- | --- | --- | --- | --- | --- | --- | --- | --- |
|  | **CCR5** | | **RRV** | | **RFHV** | | **RLCV** | |
|  | Average | C.V.% | Average | C.V.% | Average | C.V.% | Average | C.V.% |
| 1,000,000 | 1,030,000 | 11.2 | 1,050,000 | 8.22 | 1,080,000 | 7.56 | 1,080,000 | 12.2 |
| 100,000 | 96,800 | 10.7 | 104,000 | 9.07 | 101000 | 8.33 | 109,000 | 11.4 |
| 10,000 | 9,670 | 14.1 | 9,860 | 11.4 | 10,100 | 9.67 | 11,000 | 23.6 |
| 1,000 | 923 | 18.5 | 836 | 12.1 | 936 | 11.5 | 677 | 27.7 |
| 100 | 98.8 | 31.9 | 107 | 24.3 | 114 | 23.7 | 94.9 | 24.3 |
| 10 | 12.8 | 43.4 | 12.7 | 43.0 | 13.4 | 28.5 | 12.1 | 46.6 |
