# Supplementary material for: Gammaherpesvirus infection and malignant disease in rhesus macaques experimentally infected with SIV or SHIV
Source: PLoS Pathog. 2018 Jul 12;14(7):e1007130. doi: 10.1371/journal.ppat.1007130 (PMC6042791; doi:10.1371/journal.ppat.1007130)
Supplement: S3 Table — (DOCX) [file ppat.1007130.s006.docx]

Table S3 Primary Antibodies and working conditions used in IHC

| **Antibody** | **Clone** | **Starting concentration** | **Used Dilution** | **Vendors** |
| --- | --- | --- | --- | --- |
| LMP1 (EBV) | CS1-4 | 230,000 μg/mL | 1:100 | Dako |
| EBV Nuclear Antigen | 0211(6F9/60) | 0.1mg/mL | 1:50 | Novus |
| ORF73/ Anti-HHV8 LNA-1 | LN53 | 100 μg/ml | 1:150 | Advanced Biotechnology Inc. |
| 3D1.2 |  |  | 1:300 | Collaborator Scott Wong |
| CD20 | L26 |  | 1:1000 | Dako |
| CD3 |  | Supernatant | 1:100 | Neomarkers |
| Pax5 | C-20 | 100 μg/ml | 1:2000 | Santa Cruz |
| Vimentin | V9 | 20-35 μg/mL | 1:500 | Sigma |
| Collagen I | Col-I | 6300 μg/ml | 1:1000 | Sigma |
| Collagen III | FH-7A | 1200 μg/mL | 1:500 | Sigma |
| a-smooth muscle Actin | GR70237-1 | 400 μg/ml | 1:5000 | Abcam |
| Desmin | DE-R-11 | 45 μg/mL | 1:600 | Leica |
| Bcl-6 | PG-B6p | 212 μg/ml | 1:200 | Dako |
| Bcl-2 | 124 |  | 1:400 | Dako |
| c-Myc | Y69 |  | 1:500 | Abcam |
| Ki-67 | SP6 | Supernatant | 1:600 | Neomarkers |
